# Can Wide Consultation Help with Setting Priorities for Large-Scale Biodiversity Monitoring Programs?

**DOI:** 10.1371/journal.pone.0113905

**Published:** 2014-12-19

**Authors:** Frédéric Boivin, Anouk Simard, Pedro Peres-Neto

**Affiliations:** 1 Département des Sciences Biologiques, Université du Québec à Montréal, Montréal, Québec, Canada; 2 Ministère des Forêts, de la Faune et des Parcs, Québec, Québec, Canada; University of Waikato (National Institute of Water and Atmospheric Research), New Zealand

## Abstract

Climate and other global change phenomena affecting biodiversity require monitoring to track ecosystem changes and guide policy and management actions. Designing a biodiversity monitoring program is a difficult task that requires making decisions that often lack consensus due to budgetary constrains. As monitoring programs require long-term investment, they also require strong and continuing support from all interested parties. As such, stakeholder consultation is key to identify priorities and make sound design decisions that have as much support as possible. Here, we present the results of a consultation conducted to serve as an aid for designing a large-scale biodiversity monitoring program for the province of Québec (Canada). The consultation took the form of a survey with 13 discrete choices involving tradeoffs in respect to design priorities and 10 demographic questions (e.g., age, profession). The survey was sent to thousands of individuals having expected interests and knowledge about biodiversity and was completed by 621 participants. Overall, consensuses were few and it appeared difficult to create a design fulfilling the priorities of the majority. Most participants wanted 1) a monitoring design covering the entire territory and focusing on natural habitats; 2) a focus on species related to ecosystem services, on threatened and on invasive species. The only demographic characteristic that was related to the type of prioritization was the declared level of knowledge in biodiversity (null to high), but even then the influence was quite small.

## Introduction

Human activities spanning from regional (e.g., deforestation) to global levels (e.g., climate change; CC) have caused global declines in biodiversity [1]. This situation has led to a wide agreement on the need of establishing biodiversity monitoring programs motivated, among other goals, by the need to detect the impacts of disturbances on the biota, to evaluate if ecosystems are converting to undesirable states and to judge whether conservation actions are fulfilling their objectives [2]–[6]. Balmford *et al.* [2] also argued that a better knowledge of biodiversity trends and their underlying causes will make it possible to emphasize the maintenance and even the restoration of decayed ecosystems functions rather than solely focus on slowing biodiversity decline. This has led the government of the province of Québec, Canada, to established strategies for the development of a biodiversity monitoring program as part of an action plan aimed at finding ways to mitigate the effects of CC on several issues, notably on biodiversity (“Plan d’action 2006–2012 sur les changements climatiques”).

Despite several claimed benefits, biodiversity monitoring programs are also heavily criticized for being objectives in themselves rather than tools to serve larger purposes. In many situations it is unclear either how monitoring programs can serve as decision making tools or generate answers to relevant questions [7]–[9]. This situation can create frustration as people endorsing biodiversity monitoring often find that they do not fulfill their expected goals and needs [10], especially given that different stakeholders (e.g., scientist, land managers, industrials, conservationists) can have different views on what should be prioritized [8]. Therefore, if a monitoring program is designed by a single stakeholder, one should not expect that it may fulfill the goals of other interested parts [11].

To avoid such problems, it can be tempting to opt for monitoring programs having a very large coverage in terms of biodiversity measures and goals in hope to please everyone (the ‘‘laundry list” approach, sensu [12]). However, budget and time constraints often lead to focus in a few elements at the expense of other relevant components (e.g., measuring ecosystem services versus precise community compositions). Hence, the design of biodiversity monitoring programs consists in gauging and prioritizing different tradeoffs, often involving difficult decisions and abandoning elements of interests [13]. One approach is to identify and consult stakeholders to help with these choices [14], [15], thus favoring consensus and collaboration among interested parts to increase the chances of long-term success of the program [11]. Consultation processes in which stakeholders and experts are involved may, among other things, provide a certain level of ecological and management certainty; i.e., if a good majority considers an element important, it is likely relevant [16]–[18] even if from the point of view of assuring political support.

Such consultation is particularly important for a region such as the province of Québec (Canada) characterized by a vast territory (∼1.6 million km^2^) with a relatively small population (7 million) concentrated into a very small portion of its territory. This situation translates into high monitoring costs related to transportation of personnel and equipment into isolated areas. High monitoring costs imposes constraints on what can be measured, and when and where to monitor, resulting in tradeoffs between potential monitoring designs. Therefore, as part of a working group involving academics and government partners aiming at putting in place a provincial-wide biodiversity monitoring program in relation to CC in Québec (project CC-Suivi; http://bit.ly/1fJr4xK), we developed a survey to identify priorities and associated targets. The survey was sent to a large body of stakeholders for two main reasons: 1) avoid restraining consultation to a small group with a potentially narrow view of the benefit-to-risk ratio of several important decisions (see questionnaire in the methods section); 2) provide a greater level of legitimacy regarding decisions that may be often interpreted as arbitrary during the planning phase of such programs. In our survey, we defined that the main objective of the monitoring program was to generate information to be used in decision making about conservation and management in the context of CC adaptation. To our knowledge, this is the largest consultation aimed at establishing monitoring priorities for a biodiversity program.

## Methods

### 2.1. Study area

The North-South axis is the main environmental and biological gradient in Québec and stretches from tundra dominated by herbaceous species (North) to temperate broadleaf forest dominated by maples (South). The East-West axis is mainly organized around a precipitation gradient (dryer in the West) which influences fire frequency, creating a landscape of younger forests in the West [19]. Of the 1.6 million km^2^ of the province, roughly half is forested (2% of the world forests), 20% consist of taiga and 24% tundra. Within these terrestrial ecosystems, about 10% are wetlands mainly consisting of bogs and fens located in the boreal and arctic regions [20]. The province has 4 500 rivers and half a million lakes, together accounting for 3% of the world's freshwater reserve. Protected areas represent 9.1% of the territory, but this percentage falls to about 2.5% when only considering the southern region of the province [21].

The province is sparsely populated with the vast majority of the population living in the southern part of the province near the St-Lawrence River (half reside in the metropolitan area of Montreal). Agriculture is important in the South and covers 14 000 km^2^. The exploitation of natural resources, mainly through mining and forestry, is a major component of the provincial economy (forestry directly employs 60 000 people and account for 10% of the provincial export revenues [22]). Hunting, fishing and tourism are also important economic activities in rural areas. Consequently, forest trees, game (e.g., cervids) and sport fishes (e.g., salmonids) are currently the most monitored species groups, and monitoring is mostly concentrated South of 51° North, the northern limit set for forest exploitation. There are currently 545 species listed at risk under the provincial jurisdiction and more than 50% of the species at risk are located in southern Québec [23].

### 2.2. Initial consultation

In order to define the key components of the biodiversity that should be targeted by a provincial-wide monitoring program, our initial approach was based on having a series of meetings with a reduced group of experts in biodiversity from both the academic and government sectors. Halfway along this process, it became clear that we were having major issues in generating among ourselves a list that would have a strong consensus in terms of prioritization and possibly support at the government level. Therefore, we established that a much broader consultation was needed to gather the opinion of a larger group of stakeholders in order to establish priorities and procure a greater level of legitimacy to the intended monitoring program. The 13 survey questions (see below) were produced in subsequent meetings by combining and refining a first set of 142 questions that was provided by 35 professionals working on biodiversity-related issues in provincial universities, governmental institutions, NGOs and private companies. The survey was made available in both French and English (the English questionnaire is available in S1 Appendix while the French questionnaire is available via request to the authors). The official provincial language is French, but English is used by a part of the population. Both versions were used in a pre-survey format taken by a reduced number of respondents (biologists and non-biologists) prior to consultation to ensure that questions were clearly understood. Answers from the pre-survey are not reported here.

### 2.3. Survey overview

The survey consisted of 10 demographic questions (e.g., age, profession; see S1 Appendix) and 13 multiple choice involving prioritization issues. Our goal was to motivate an active choice regarding prioritization schemes involving cost tradeoffs (i.e., if you do more of something, you will have reduced funding for something else). Therefore, we only allowed respondents to select between one or two choices across all questions, even though other options could have been also seen as important. It was identified at the beginning of the survey that the low number of choices was done intentionally and served to represent the real difficulties that monitoring programs face while prioritizing under limited funding. Even if funding would not be limiting, certain aspects would always tradeoff (i.e., one cannot logistically monitor every species across all ecosystems every year).

The first set of questions (1 and 2) related to the localization of monitoring sites and aimed at determining where their distribution should be along a North-South or East-West axis (see Study area section above for the importance of these axes within the province) and the principal land or ecosystem types to monitor. As interactions between CC and other human activities are expected, we also asked (question 3 and 4) what anthropogenic perturbations other than CC should be considered and if sites should be positioned in pristine or human-impacted areas.

The second series of prioritization questions (5 to 9) were related to biodiversity indicators and dealt with the type of species to target, the type of geographic distribution that the target species should have (i.e., small or large in range) and the amount of information to record for each indicator. Two of these questions (8 and 9) directly targeted tradeoffs (i.e., measuring more of some elements implies measuring less of others). These questions implied that for a given budget, increasing monitoring geographic or taxonomic coverage has to come at the expense of having less information for each element, hence increasing the probability of detecting changes but decreasing our capacity to find the cause (e.g., organisms reduced in abundance locally versus organisms dispersed). Another question (10) asked whether a monitoring program should focus on generating scientific knowledge or serving as an alarm system to report biodiversity changes. By this question, we referred to warning signals likely being generated more rapidly (e.g., measuring multiple elements with fewer details to determine if certain biodiversity elements are changing) at the expense of fully understanding their causes, i.e., build scientific knowledge would take more time. The final two questions (12 and 13) asked whether respondents thought that euthanizing organisms for future research was acceptable and if the respondents would be willing to volunteer half a day per year to a citizen science project.

### 2.4. Administration of the survey

Stakeholder groups identified as targets included:

- Government researchers and managers from the three environmentally-related provincial ministries (natural resources and wildlife - *Ministère des Ressources naturelles et de la Faune*; sustainable development, environment and parks - *Ministère du Développement durable, de l'Environnement et des Parcs*; agriculture, fisheries and food - *Ministère de l'Agriculture, des Pêcheries et de l'Alimentation du Québec*), and provincial offices of four federal agencies (Environment Canada, Fisheries and Oceans Canada, Canadian Forest Service, Agriculture Canada),
- Professors from departments of biology, environment, land management, geography, agriculture, communication, philosophy, politics and sociology from all provincial universities,
- Employees and volunteers of about 140 non-governmental organizations (NGO) active in environment,
- Employees of provincial watershed management bureaus,
- Industrial employees in the environmental sector (electricity, forestry, gas, petroleum and mining),
- Employees of consulting firms specialized in impact assessment,
- Elected officials (city mayors, and federal and provincial deputies),
- Pre-university biology teachers,
- Managers of “controlled exploitation zones” (“*Zones d'Exploitation Contrôlée*” or “ZEC”; organizations in charge of planning, organizing and controlling the exploitation, conservation and management of fauna in the province),
- Farmers and
- First Nation members.

The survey was not aimed at the population at large, but rather targeted individuals based on two non-exclusive goals, (i) gathering input on monitoring priorities from individuals with different biodiversity and land management backgrounds and (ii) make the initiative known within stakeholder groups. Hence, like in several other surveys (e.g., [24], [25]), participants were not randomly chosen but belonged to specific demographic groups, which included potential participants with variable biodiversity and land management knowledge. The invitation to participate in the survey was sent to this pool of potential participants by email either directly (we built large lists based on visiting internet sites of the various organizations) or through associations (e.g., *Réseau québécois des groupes ecologists [RQGE] – Quebec's network of ecological groups*, an organization with the objectives of linking Québec's environmental groups, sharing information among them and representing them at the political level; http://www.rqge.qc.ca/). Given that in many cases we were not able to contact individuals directly (e.g., government agencies mandating that the survey be sent by them to their employees, RQGE forwarding the email to ecological groups contacting their members), it was not possible to estimate response rates. Although some may not see that as ideal when the goal is to estimate how representative the survey was, our goal was to gather information on prioritization choices from as many participants as possible and make the initiative known. Given that the contact information of our demographics was not easily available (e.g., employees of impact assessment companies), we proceeded in a way that increased the odds of reaching as many potential participants as possible (see [25] for a similar approach). This approach also allowed us to contact groups that we could not have reached otherwise (e.g., government agencies do not provide email lists of their employees). The invitation included a short description of the project and links to the English and French versions of the survey (hosted on the web-based application SurveyMonkey; http://www.surveymonkey.com). Invitations were sent at the end of September 2011. A six weeks period was allowed and a reminder was sent two weeks prior to the final closure date.

### 2.5. Statistical analyses

Values are reported in the form of number of respondents that selected a particular option for each question. Correspondence analysis [26] estimates the relationship among categorical variables and was used to identify possible links among priorities. In this case, we created a matrix in which each respondent was a row and questions as columns (e.g., question 1 had 4 possible choices and was represented by 4 columns, question 2 had 3 possible choices and was represented by 3 columns, and so on; data are reported in S2 Appendix). The correspondence analysis was run on an indicator matrix (respondents by prioritization categories) that identified with 1s the options selected by each respondent and 0 otherwise. The amount of explanation of each axis can then be used as an indicator of how related the prioritization categories are [27].

A matrix was produced to represent respondent profile in a similar way in which the prioritization matrix above was produced (data are reported in S2 Appendix). In order to determine whether prioritization was related to the respondents' profiles, we applied a multivariate regression tree [28] where the priority matrix was used as the response (dependent) matrix and the profile matrix was used as the predictive matrix. A 10-fold cross validation procedure was repeated 100 times and the final retained classification tree was the one with the lowest cross-validation error. All analyses were performed in the R environment [29] using the *vegan* [30] and *mvpart* [31] packages.

### 2.6. Ethics statement

We were granted a written approval from the “Comité institutionnel d'éthique de la recherche avec des êtres humains” (human research ethics committee) of the Université du Québec à Montréal before conducting the survey (approval no. 709991).

## Results

### 3.1. Profile of respondents

621 individuals completed the survey. The majority of the respondents were employees of governmental agencies (38%) and universities (21%). 85% of the respondents had a university diploma (24% bachelor, 36% masters and 25% PhD) and 74% had formal training in a biodiversity-related field (biology 40%, environment 13%, geography 8%, forestry 7%, ecology 6%) while the rest of the participants came from other fields, such as education (2%) and politics (5%). The majority of participants declared having a good (58%) or excellent (26%) knowledge of biodiversity and the remainder 16% declared low or no knowledge.

The first three axes of the correspondence analysis explained little variation (i.e., 4.1%, 3.9% and 3.8%, respectively) suggesting that the answer given to a particular question was not a good predictor of answers to other questions, thus, indicating a good level of separation between criteria for establishing a monitoring program. The multivariate regression tree, based on the minimization of the cross-validated relative error [28], identified “declared knowledge about biodiversity” as the only significant predictor of how respondents prioritized different monitoring schemes. However, note that the MRT explained next to no variation (R^2^ = 0.01).

### 3.2. Questionnaire

**Figure 1:**
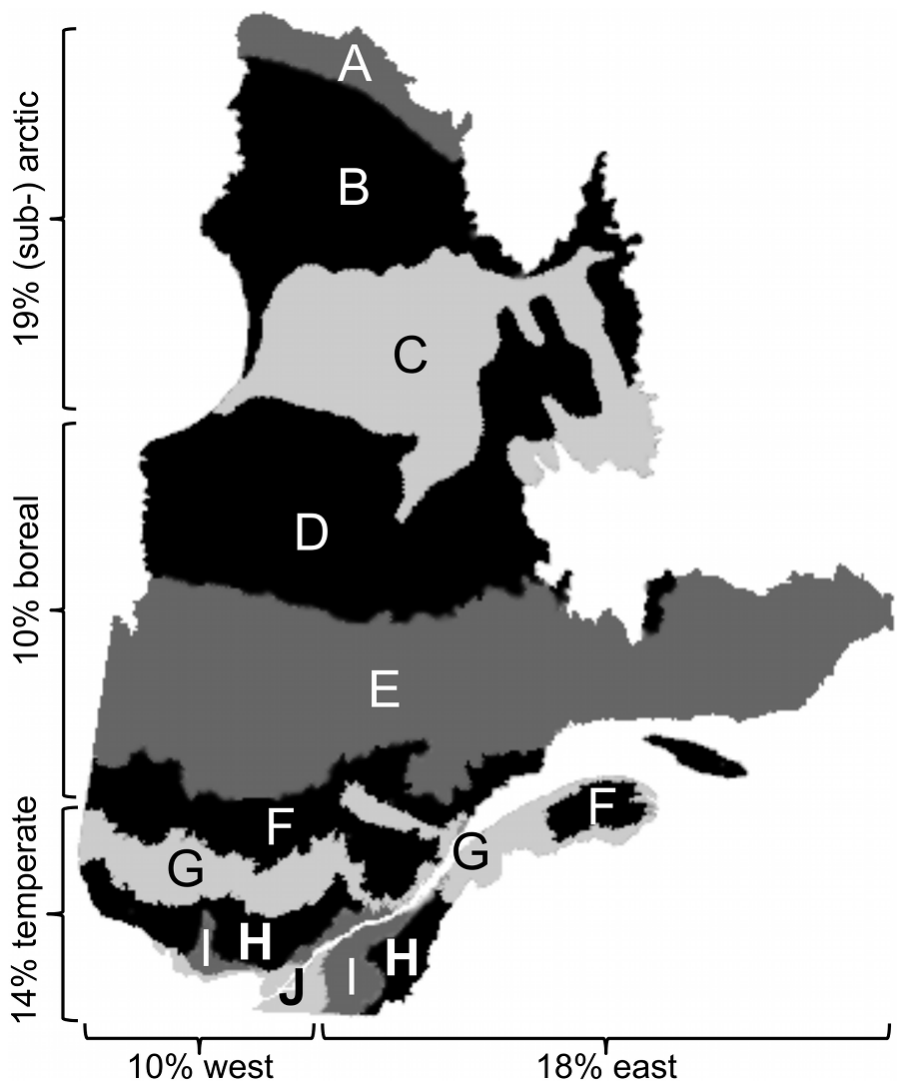
Geographic regions to prioritize for the monitoring of the effect of climate change for the province of Québec, Canada, along North-South and East-West axis as chosen by the participants to a survey. The percentage for each geographic region is indicated along the province sketch. Note that these percentages do not sum to a hundred as 57% (North-South) and 71% (East-West) of the participants answered that no region should be prioritized. Each geographic region comprises several bioclimatic domain delineate by the different shades of grey. The (sub-) arctic region (North) includes, A - herbaceous tundra, B- shrub tundra and C - forest tundra (taiga); the boreal region (center) comprises, D - black spruce – lichen forest, E - black spruce – moss forest and F - fir – white birch forest; the temperate region (South) contains, G - fir – yellow birch forest, H - maple – yellow birch forest, I - maple – basswood forest and J - maple – hickory forest. The East-West axis (not detailed on the map) is mainly centered on a decreased precipitation gradient from East to West causing an increase in fire frequency and a decrease in average forest age. The map is modified from http://www.mrn.gouv.qc.ca/forets/connaissances/images/zonesBioClim.gif, consulted April 2013.

Fig. 1 presents a map identifying how participants prioritized different regions. The majority chose the no priority option for both the North-South (57%) and the East-West (72%) axis. Among respondents who favored a region along the North-South axis, 19% chose to focus in the North (taiga and tundra), 14% in the South (the temperate region) and 10% in the center (the boreal region). Among those who favored a region along the East-West axis, 10% and 18% opted for East and West, respectively (Fig. 1).

**Figure 2:**
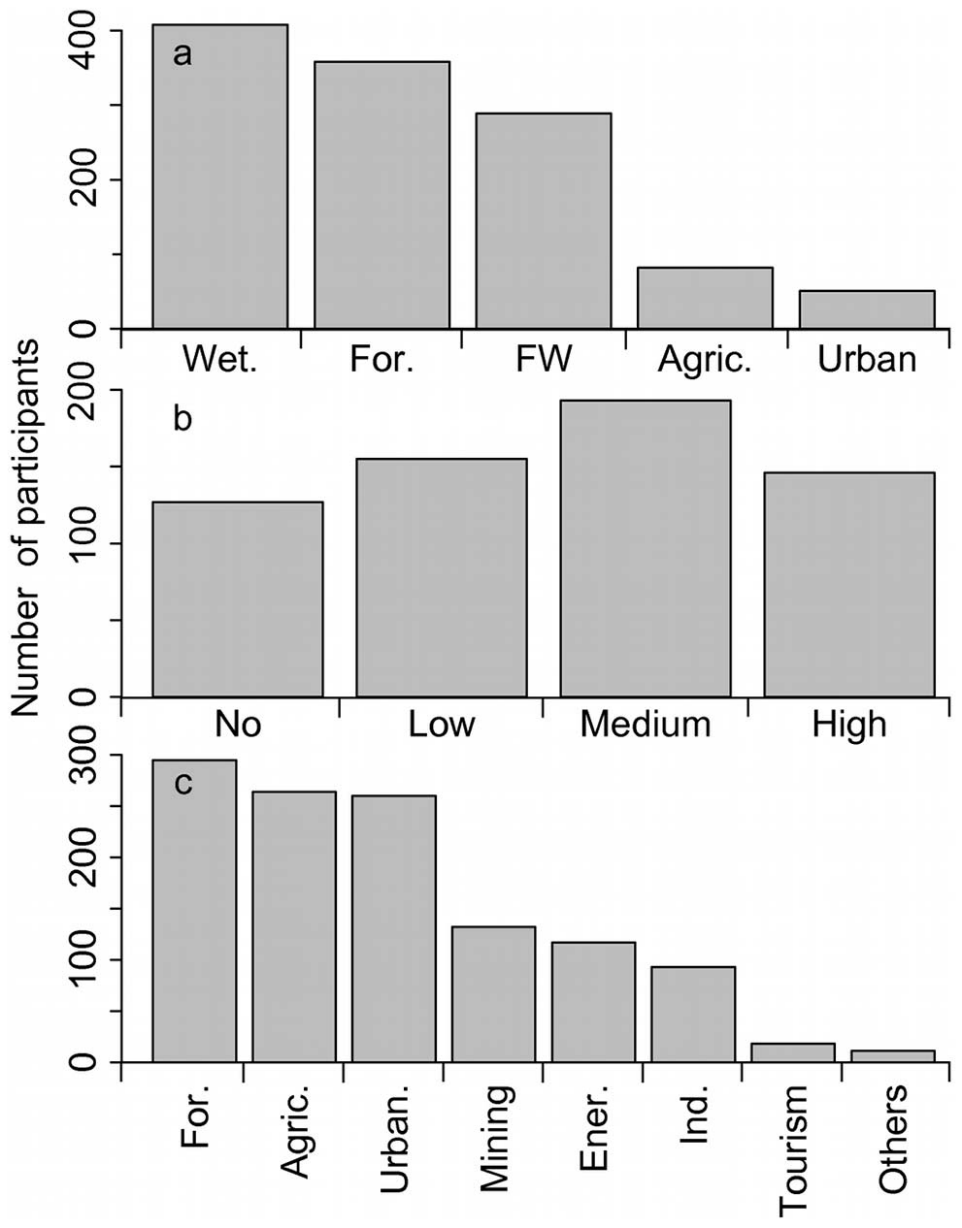
Number of participants choosing each answer for three questions related to sites. a) What do you think are the ecosystems of greatest priority for monitoring biodiversity in the context of climate change (one or two options)? Abbreviations; Wet.  =  Wetlands, For.  =  Forests, FW.  =  Freshwater systems, Agric.  =  Agricultural land. b) In your opinion, areas with what level of human disturbance should be the priority for monitoring biodiversity in the context of climate change (one option)? c) If there is biodiversity monitoring in areas with some human impact, what types of activities do you think should be prioritized for monitoring (one or two options)? Abbreviations; For.  =  Forestry, Agric.  =  Agriculture, Urban.  =  Urbanization, Ener.  =  Energy, Ind.  =  Industry.

When asked to prioritize one or two types of ecosystems to monitor, the majority of respondents selected one of the natural ecosystems (wetlands 66%, forests 58%, freshwaters 47%) while a minority chose anthropic environments (urban 8%, agricultural areas 13%; Fig. 2a). Respondents choosing the anthropic environments were higher among those with low or no knowledge of biodiversity (urban 16%, agricultural areas 20%). The next question asked if the monitoring program should focus on areas with high, medium, low or no (pristine) human disturbance. Participants where divided on the matter as 24% chose high, 31% medium, 25% low and 20% no disturbance (Fig. 2b). 39% of the participants with low or no knowledge of biodiversity prioritized monitoring in areas with high disturbance. Respondents were then asked to select among disturbance other than CC that should also be targeted (Fig. 2c). Most participants opted between forestry (48%), urbanization (42%) and agriculture (42%); these proportions were even higher for participants with excellent knowledge of biodiversity (58, 48 and 47%, respectively). Three other disturbances (mining, other industries and energy production) where chosen by respectively 21%, 19% and 15% of participants, while only 3% chose tourism as a disturbance to consider.

**Figure 3:**
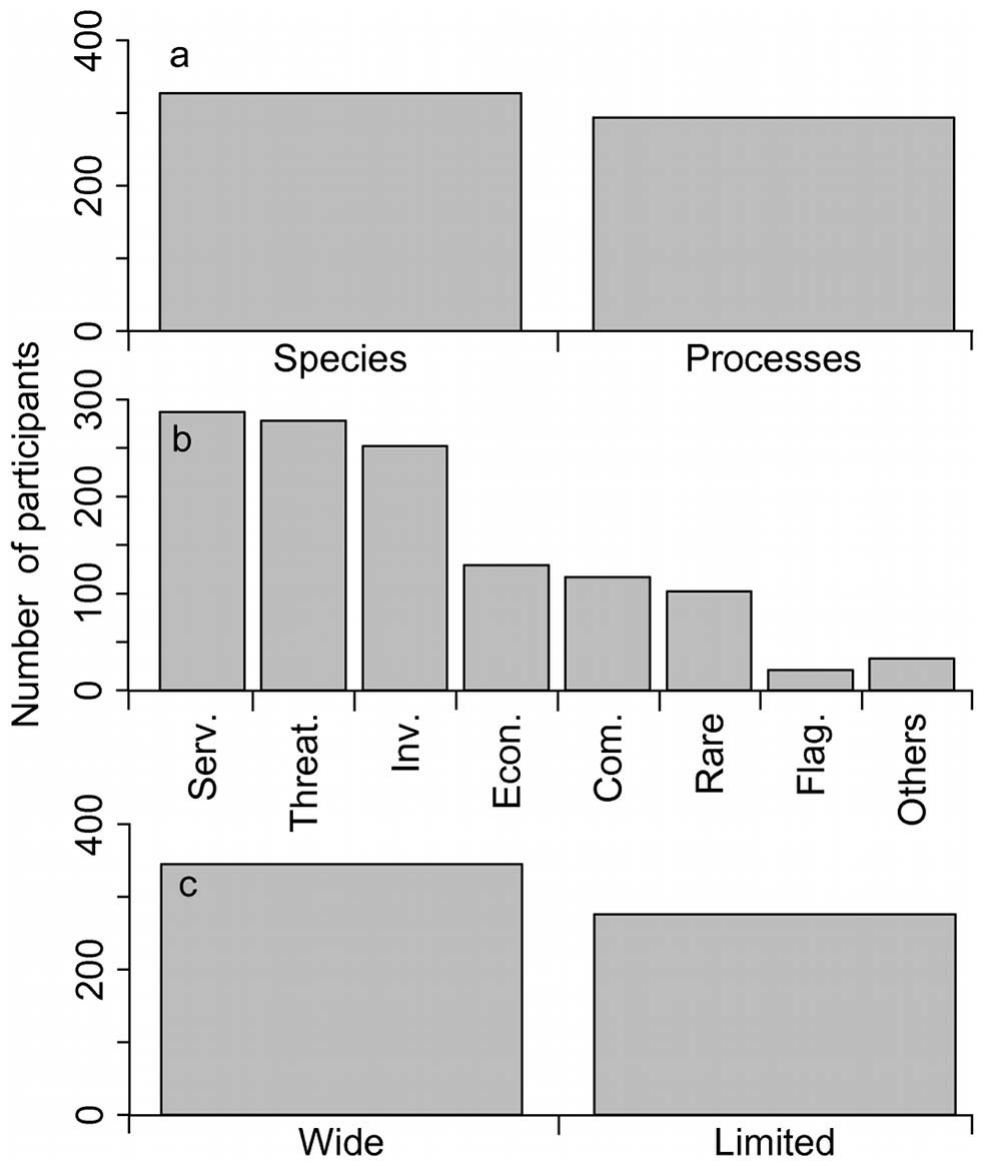
Number of participants choosing each answer for three questions related to indicators. a) In your opinion, should the biodiversity monitoring program focus more on gathering data at the level of species, such as abundance or distribution, or at the level of ecosystem processes, such as productivity and decomposition (one option)? b) What kind of species do you believe the biodiversity monitoring program should prioritize (one or two options)? Abbreviations; Serv.  =  Species providing important ecological services, Threat.  =  Endangered and threatened species, Inv.  =  Invasive and/or harmful species, Econ.  =  Economically important species, Com.  =  Common, Rare  =  Rare, but not endangered or threatened, Flag.  =  Emblematic species. c) Do you think that efforts to monitor biodiversity in the context of climate change should focus more on species with a wide distribution across Québec or species with a limited distribution representative of the different regions of Québec (one option)?

Respondents were equally split when asked to choose whether the monitoring program should focus on species or on ecosystem processes (e.g., productivity, pollination), as 53% versus 47% answered species over process (Fig. 3a). The next question asked to choose one or two types of organisms to target (e.g., rare, common, economically important; Fig. 3b) rather than specific taxa. Three categories were prioritized, namely species providing important ecosystem services (46%), endangered and threatened species (45%), and invasive or harmful species (41%). Other priorities were economically important wild species (21%), common species (19%), and rare but not endangered species (16%). Respondents with low or no knowledge of biodiversity chose economically important species in a greater proportion (28%) in contrast with those having excellent knowledge (16%). Emblematic species were prioritized by only 3% of the participants. When asked whether participants thought that the monitoring program should focus on species with a wide or limited geographic distribution, 56% chose a wide distribution (Fig. 3c).

**Figure 4:**
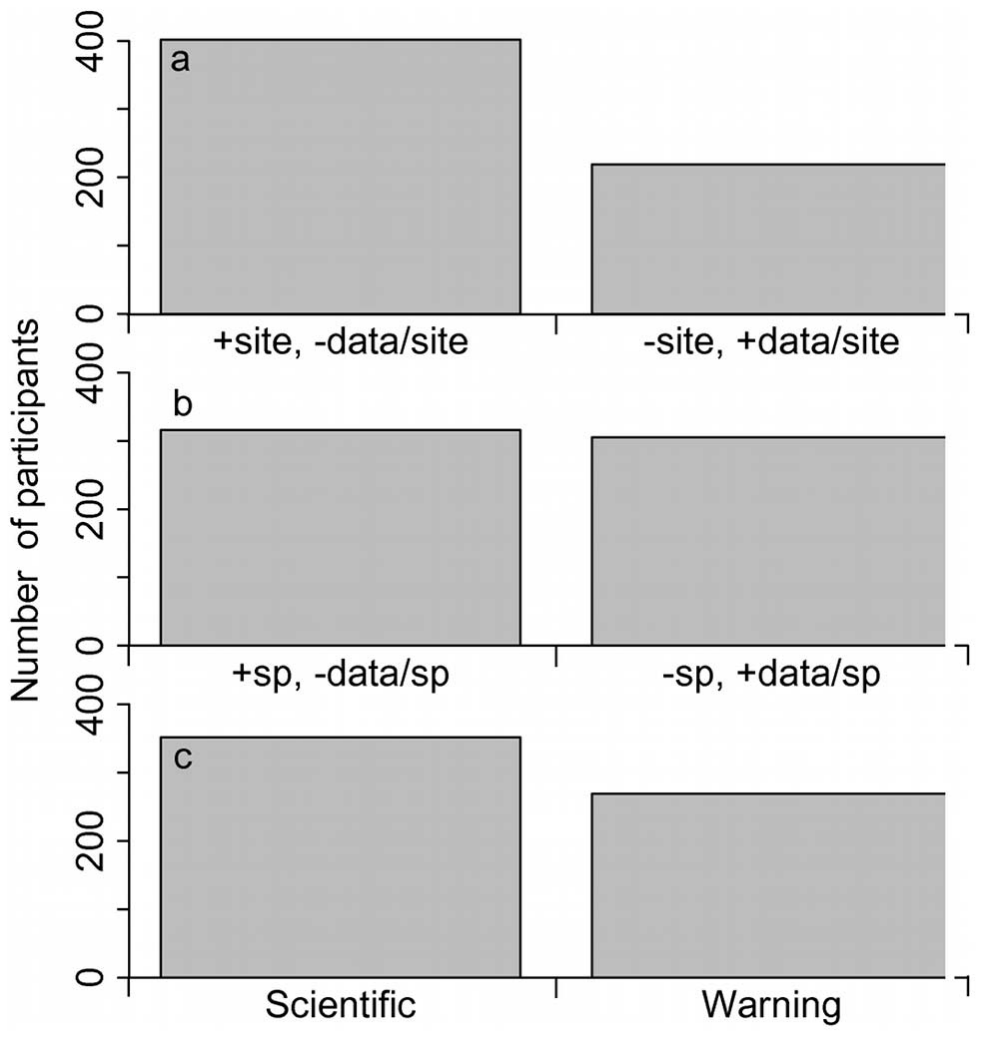
Number of participants choosing each answer for three questions related to direct tradeoffs. a) Do you think that the biodiversity monitoring program should include a larger number of sites but with less data collected per site or a smaller number of sites but with more data collected per site (one option)? b) At each biodiversity monitoring site, do you think the focus should be on measuring more variables per species, but for a smaller number of species, or on measuring fewer variables per species, but for a larger number of species (one option)? c) In your opinion, should the monitoring program be designed more to generate warning signals (indications of whether or not climate change is or has been impacting biodiversity), or more to build a scientific understanding (information about how biodiversity is being affected by and responding to climate change) (one option)?

The first question tackling tradeoffs asked participants if it would be better to have a monitoring program with more sites but less data per site or less sites with more data with the former being selected by 65% (Fig. 4a). Participants were divided nearly equally (51% and 49% respectively; Fig. 4b) when asked if it was better to follow more species with less data per species or the inverse. Finally, 57% of the respondents indicated that it would be better to design a monitoring program based on building scientific understanding rather than on generating warning signals (43%).

**Figure 5:**
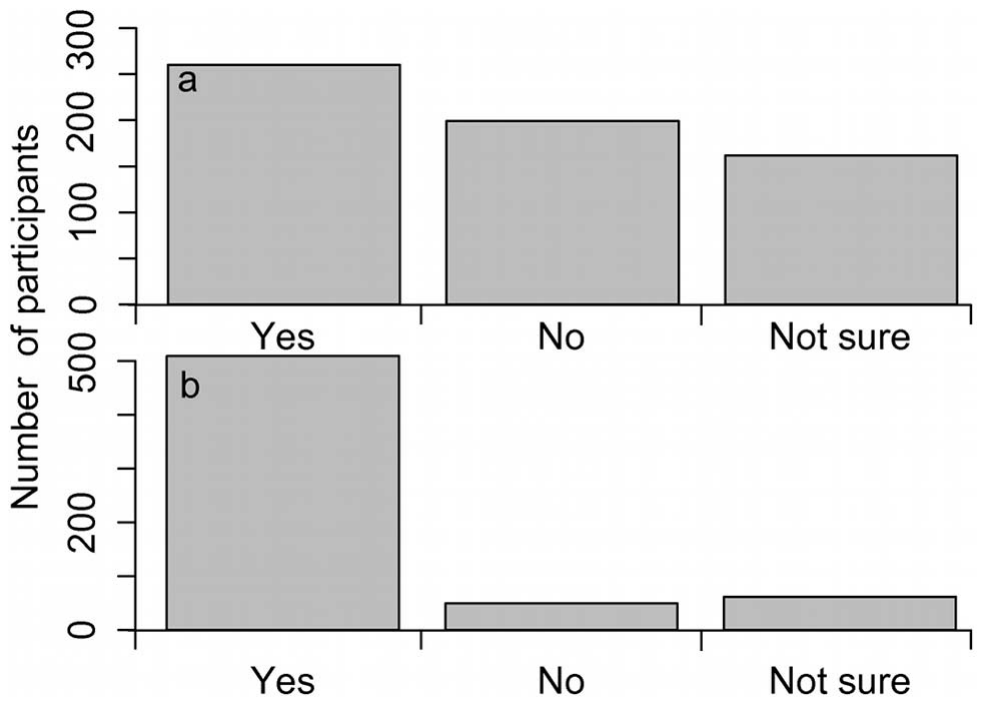
Number of participants choosing each answer for two questions related to ethics and citizen participation. a) Do you believe that species samples should be euthanized and put in scientific collections to be used for research at a future time when better technology and more resources are available to analyze these samples (one option)? b) Would you be willing to devote a half-day per year or more of your personal time to participate, as a citizen scientist, in biodiversity monitoring related to climate change (one option)?

Most respondents indicated that species samples could be euthanized and kept for future research at a future time when better technology and more resources are available to analyze them (42%), 32% were against and 26% had no opinion on the matter (Fig. 5a). Finally, 82% of the respondents indicated that they would be willing to devote a half-day to participate in biodiversity monitoring initiatives related to CC, while 8% said no and 10% were not sure (Fig. 5b).

## Discussion

CC and biodiversity loss are among the greatest challenges that humanity will have to face in the next century [1]. To face these challenges, there is a pressing need to increase our understanding of both these issues and also the links and feedbacks between them, a task for which monitoring can serve as a valuable tool [32]. However, considering that budget for such an endeavor is limited, monitoring all aspects of biodiversity is not a sustainable option and certain elements must be prioritized over others. Determining monitoring priorities is well recognized as challenging [6], [8], [33] and the results of our study reinforce this view given the lack of consensus among respondents for 11 out of the 13 questions. Note that as in other studies (e.g., [25]), one limitation from our survey is that we were not capable of estimating the potential number of respondents within each demographic class in order to produce a stratified selection of individuals across classes (see method). However, as the lack of consensus does not appear related to demographics (based on the MRT analysis), potential bias that could arise from this limitation seems insignificant.

Several studies have used opinion-based assessments to identify crucial questions that should be tackled for the conservation of biodiversity [24], [34], [35], though few underwent a prioritization process (e.g., [36]). For example, Maddock and Samways [37] sought opinion from professional conservationists (with at least 5 years of experience) and obtained a fairly clear consensus about important areas and priorities for conservation. Their larger consensus may have been obtained due to the fairly homogenous group consulted. Other conservation questions prioritization exercises also obtained fairly clear rankings by contacting participants from demographics similar to ours (policymakers, scientists and managers) [30], [42], though Human and Davies [36], who consulted a very diverse stakeholder demographics through workshops to identify research priorities for a coastal region in Australia, found, as we did, that there was poor consistency.

One of the reasons put forward to explain the difficulties in implementing monitoring programs is that while collaboration among stakeholders is essential for biodiversity monitoring to succeed [11], stakeholders have different opinions about what should be measured [8]. However, like others [25] (but see [38]), we did not find that differences in prioritization were related to the respondents' demographic characteristics. In fact, the only factor that could set apart participants was their declared knowledge of biodiversity, but, even there, differences were relatively small.

A number of explanations can be put forward to explain our results. For example, it has been suggested that when it comes to setting conservations priorities, choices can strongly differ on a personal level based on perceptions and values [39]. Another possibility is that a low level of general knowledge of the science of monitoring and of the effects of CC on biodiversity might have been responsible for a lack of clear priorities [36]. Whatever the underlying reason, if convergence of opinions from specialists is considered as an indication of ecological certainty [16]–[18], we were not able to determine a clear set of priorities based on our survey. This does not bode well with the idealistic view of finding a consensus for the design of monitoring programs and may explain why such programs are often controversial and polarizing (see as an example the debate over the design and utility of the Alberta Biodiversity Monitoring Program, one of the largest monitoring program in the world [7], [40], [41]).

Notwithstanding these observations, the lack of consensus could be considered as the result of expert consultation directing towards a too broad set of goals instead of a set of specific targets. Overall, it appears that to satisfy the large majority would require monitoring lots of different elements. However, this brings the issue raised earlier regarding the dangers of using a “laundry list” of indicators within monitoring programs [12]. There is a thin line between favoring a wide variety of aspects within monitoring programs and being scattered over too many aspects resulting in no meaningful findings [8] in addition to the elevated costs that could eventually jeopardize the long term persistence of the monitoring program. For instance, all natural ecosystems were similarly valorized, as well as several types of disturbances and species types. Moreover, most participants clearly preferred a monitoring program covering the entire provincial territory, and not just concentrated near populated centers (i.e., in the South for Québec province), as commonly observed in most monitoring programs worldwide. Interestingly, in addition to the 57% of respondents that indicated that the monitoring program should cover the North-South axis entirely, 19% of them would like the monitoring program to focus on the North where no monitoring is currently done. Considering logistics and financial constraints associated to northern monitoring, this would involve fewer sites or biodiversity indicators.

Another interesting impasse is that the monitoring of both ecosystem processes and species appeared nearly identical. Although, priorities for monitoring one of these elements does not preclude the other (both are intimately linked and can be followed at the same time [42]), emphasizing either one would greatly influence the development of questions that the program will be able to answer and, ultimately, its design. Presently, the (possible) effects of CC on species are better known than its effects on ecosystem processes and services [43] due in part to the fact that data on services and processes are rare [44]. The development of new monitoring programs may provide an opportunity to close this knowledge gap. The interest in prioritizing ecosystem processes seems strong given that the majority of respondents prioritized the monitoring of species providing important ecosystem services (regardless whether ecosystems processes or species were chosen as the main target).

The aspect that had the stronger consensus (82%) related to the interest of participants in devoting half a day per year with citizen science activities associated to biodiversity monitoring. Programs using citizen participations are more and more numerous worldwide [45], [46] and even though there are difficulties linked to the analysis of data from citizen sciences initiatives (e.g. differences in citizens' capability to identify organisms [47]), the large quantity of information that can be gathered may well compensate for these issues [48]. Although it could be argued that individuals who answers surveys about biodiversity monitoring programs are those more inclined to participate in citizen science initiatives, the willingness of such a large proportion of respondents to invest personal time may be a sign that there is room for growth for such initiatives.

## Conclusions

It is likely that all aspects of biodiversity will be affected by CC. Although some of these aspects will probably be affected faster or more critically than others, there are valuable scientific or political arguments to be made to monitor practically all of these aspects. Individual interests and priorities, an assertion supported by our survey, largely influence what is optimal to monitor given that respondents were largely divided across different priorities. Therefore, although it is important to try to reach agreement among stakeholders while designing monitoring programs, it appears that this may be very difficult to achieve, especially when resources are limited and the number of indicators that can be followed is small. As such, biodiversity monitoring designers have to be prepared to make difficult decisions that will not necessarily lead to a consensus.

## Supporting Information

S1 Appendix The English version of the survey provided to the participants including the introduction text.
(DOCX)

S2 Appendix Survey results separated into two tables. The table “Demographics” contains answers from the 10 demographic questions in dummy format (1 yes, 0 no). The table “Priorities” contains answers from the 13 priority questions in dummy format. Questions can be found in S1 Appendix.
(XLS)
