# Supplementary material for: Can Wide Consultation Help with Setting Priorities for Large-Scale Biodiversity Monitoring Programs?
Source: PLoS One. 2014 Dec 19;9(12):e113905. doi: 10.1371/journal.pone.0113905 (PMC4272257; doi:10.1371/journal.pone.0113905)
Supplement: S1 Appendix — The English version of the survey provided to the participants including the introduction text. (DOCX) [file pone.0113905.s001.docx]

**Appendix S1.** English Version of the Survey Provided to the Participants.

The text between lines was on the first page of the survey and served as an introduction.

**Introduction**

As part of Québec’s action plan on climate change, the government seeks to design and implement a program for monitoring the effects of climate change on biodiversity in terrestrial and freshwater systems. The aim of the monitoring program is to generate information that can be used for decision-making about conservation and management.

A research team from the Department of Biology in the Université du Québec à Montréal (UQAM) is helping to design this monitoring program, which is described on the [project website](http://www.er.uqam.ca/nobel/r3424621/CC-Suivi/f/fr/Accueil.html.). Creating this program requires making challenging decisions about exactly what, where, and how to monitor in order to detect changes in biodiversity that are a function of climate change. The objective of this survey is to identify the priorities of a range of stakeholders from many different parts of Québec society.

**Your responses will help the research team, and in turn the government of Québec, to make key decisions about how to design this program for monitoring biodiversity in the context of climate change in Québec**.

*Please note*: Participation in this survey is anonymous. The data collected from the survey will be securely managed and accessible only to members of the research team. Survey results will be presented in a way that does not compromise the confidentiality of respondents.

*If you would like, you may forward the survey link to other colleagues to complete.*

**This survey should take you between ten and twenty minutes to complete.**

**The following is the questionnaire provided to the participants. Squares on the left indicate checkbox options, lines indicates that the participant could write is/her answer.**

**Part one: Participant information**

1. **In what city or town do you live?**

{drop down menu}

1. **How many years have you lived in Québec?**

☐ Less than 5

☐ 5-9

☐ 10-19

☐ 20-29

☐ 30-39

☐ 40-49

☐ 50 or more years

Have you lived in Québec your entire life?

☐ Yes

☐ No

1. **In what sector do you work?**

☐ Québec or Canada government

☐ Regional or municipal government

☐ University or research institution

☐ CEGEP

☐ Student

☐ Consulting

☐ Industry (Energy, mining, forestry, tourism, etc...)

☐ NGO

☐ Agriculture or aquaculture

☐ Self employed

☐ Other: _____________

1. **What is your job title?** *(Examples: farmer, technician, professor, planner)*

______________________

1. **In what field do you currently work?** *(Examples: biology, finance)*

_____________________

1. **What is the field of your last degree, if applicable?** (*Examples: geography, economics, law)*

______________________

1. **What is your gender?**

☐ Male

☐ Female

1. **What is your age?**

☐ Less than 20

☐ 20-29

☐ 30-39

☐ 40-49

☐ 50-59

☐ 60 or older

1. **What is the highest level of education that you have completed?**

☐ High school

☐ CEGEP

☐ University - Bachelor

☐ University - Masters

☐ University - PhD

☐ Professional studies

☐ Other: ______________

1. **What is your level of knowledge about biodiversity?**

☐ No knowledge

☐ Low knowledge

☐ Good knowledge

☐ Excellent knowledge

**Part two: Monitoring priorities**

As you answer the following twelve questions, please keep in mind that resources for monitoring are always finite and so it is never possible to monitor *every* variable related to *every* species in *every* ecosystem in Québec. We have limited the possible number of options that you can choose in order to identify your priorities. Each question includes space for your comments, should you want to provide any. We are asking you to respond based on your personal perspective, rather than the needs or priorities of your employer or association.

1. **For monitoring biodiversity in the context of climate change, in which geographic region(s) of Québec should efforts be focused?**
2. **North to South: Please choose one option.**

☐ North (taiga and tundra)

☐ Center (boreal region)

☐ South (temperate region)

☐ I don’t think there should be a priority (i.e., all regions are equally important).

1. **East to west: Please choose one option.**

☐ East

☐ West

☐ I don’t think there should be a priority (i.e., all regions are equally important).

Comments (optional):

1. **What do you think are the ecosystems of greatest priority for monitoring biodiversity in the context of climate change? Please choose one or two options from the list below.**

☐ Wetlands

☐ Urban

☐ Lakes and streams

☐ Agricultural

☐ Forests

Comments (optional):

1. **In your opinion, areas with what level of human disturbance should be the priority for monitoring biodiversity in the context of climate change? Please choose one option.**

☐ No disturbance (pristine)

☐ Low level of disturbance

☐ Medium level of disturbance

☐ High level of disturbance

Comments (optional):

1. It is expected that interactions between climate change and human activities will lead to an increased overall impact. **If there is biodiversity monitoring in areas with some human impact, what types of activities do you think should be prioritized for monitoring? Please choose one or two options.**

☐ Mining

☐ Urbanization

☐ Agriculture

☐ Tourism

☐ Industry

☐ Forestry

☐ Energy *(Examples: wind, hydropower)*

☐ Other: _____________________

Comments (optional):

1. **In your opinion, should the biodiversity monitoring program focus more on gathering data at the level of species, such as abundance or distribution, or at the level of ecosystem processes, such as productivity and decomposition? Please choose one option.**

*Note: With a focus on species, changes in ecosystem processes that are important to humans may not be detected. With a focus on ecosystem processes, changes in and loss of species may not be detected if they do not have a detectable impact on ecosystem processes.*

☐ Species (for example, abundance and distribution)

☐ Ecosystem processes (for example, productivity and decomposition)

Comments (optional):

1. **What kind of species do you believe the biodiversity monitoring program should prioritize? Choose one or two options, even if you prioritized ecosystem processes in question #5.**

☐ Endangered and threatened species/species at risk (*Examples: wood turtle, Bicknell thrush)*

☐ Rare, but not endangered or threatened, species *(Examples: pitcher plant, Québec emerald dragonfly*)

☐ Common species *(Examples: white tailed deer, American toad)*

☐ Economically important wild species *(Examples: Atlantic salmon, sugar maple)*

☐ Species providing important ecological services *(Examples: freshwater mussels, bumblebees)*

☐ Emblematic species *(Examples: caribou, snow goose)*

☐ Invasive and/or harmful species (*Examples: disease vectors, zebra mussel)*

☐ Other: _________________

Comments (optional):

1. **Do you think that efforts to monitor biodiversity in the context of climate change should focus more on species with a wide distribution across Québec or species with a limited distribution representative of the different regions of Québec? Please choose one option, even if you prioritized ecosystem processes in question #5.**

☐ Species with a wide geographic distribution

☐ Species with a limited geographic distribution

Comments (optional):

1. **Do you think that the biodiversity monitoring program should include a larger number of sites but with less data collected per site or a smaller number of sites but with more data collected per site? Please choose one option.**

*Note: With a larger number of sites, there would be a greater chance of detecting localized changes (due to a more thorough geographical covering), but less chance of detecting minor changes (due to less thorough sampling per site). With a smaller number of sites, there would be more chance to detect minor changes, but less chance of detecting localized changes.*

☐ Larger number of sites and less data collected per site

☐ Smaller number of sites and more data collected per site

Comments (optional):

1. **At each biodiversity monitoring site, do you think the focus should be on measuring more variables per species, but for a smaller number of species, or on measuring less variables per species, but for a larger number of species? Please choose one option.**

*Note: With a larger amount of data for a small number of species, there is a greater chance of understanding the causes of changes affecting some species, but certain changes would only be detected for the limited number of selected species. With less data per species, there is a greater chance of detecting changes that affect only a few species, but there is less of an opportunity to understand the causes of those changes.*

☐ More data per species for a smaller number of species

☐ Less data per species for a larger number of species

Comments (optional):

1. **In your opinion, should the monitoring program be designed more to generate warning signals (indications of whether or not climate change is or has been impacting biodiversity), or more to build a scientific understanding (information about how biodiversity is being affected by and responding to climate change)? Please choose one option.**

*Note: Warning signals would likely be generated more rapidly and thereby enable more rapid response, but would contribute less to a scientific understanding of the changes. Building scientific understanding would take significantly more time but would be more thorough, and the knowledge would be more transferable to other situations.*

☐ Generate warning signals

☐ Build scientific understanding

Comments (optional):

1. **Do you believe that species samples should be euthanized and put in scientific collections to be used for research at a future time when better technology and more resources are available to analyze these samples? Please choose one option.**

☐ Yes

☐ No

☐ I don’t have an opinion about this.

Comments (optional):

1. **Would you be willing to devote a half-day per year or more of your personal time to participate, as a citizen scientist, in biodiversity monitoring related to climate change? Please choose one option.**

*Note: we will not contact you if you answer “yes”. This question is strictly for the survey.*

☐ Yes

☐ No

☐ I am not sure.

Comments (optional):

**Thank you for your participation.**
